# Breastfeeding support for Australian hospital employees: a qualitative study

**DOI:** 10.1186/s13006-026-00834-9

**Published:** 2026-04-13

**Authors:** Lynne Henry, Virginia Schmied, Elaine Burns

**Affiliations:** 1 School of Nursing and Midwifery, Western Sydney University, Parramatta, Australia; 2 Women’s and Newborn Health, Westmead Hospital, Sydney, Australia

**Keywords:** Breastfeeding, Return to work, Hospital employees, Workplace support, Gender equity

## Abstract

**Background:**

Returning to paid work from maternity leave is a known barrier to achieving breastfeeding duration goals. The Australian hospital workforce operates under mandated state and local policy directives, which stipulate breastfeeding is to be promoted, protected, and supported. While breastfeeding employees’ experiences have been examined across multiple workplace sectors, little is known about how Australian hospital employees manage. It has been reported internationally that hospital employees have a high risk of any breastfeeding cessation on return to work. This study aimed to explore the lived experiences of hospital employees returning to work while breastfeeding at one hospital in Sydney, Australia.

**Methods:**

Data collection occurred from March to May 2021. All hospital employees who had returned to work maintaining breastfeeding over the previous four years were invited to participate however employees who volunteered were from nursing, medical, allied health, and administration staff positions. A qualitative interview design enabled collection of in-depth data, focused on hospital employees’ lived experiences of returning to work breastfeeding at this hospital site. Audio-recorded interviews were transcribed verbatim, coded in NVivo, and analysed into themes and subthemes using the seven-step Interpretive Phenomenological Analysis (IPA) process.

**Results:**

Thematic analysis of thirteen interview transcripts generated five themes: ‘*Continuing to breastfeed was a big concern’*,* ‘The reality of pumping at work’*,* ‘Navigating co-worker negativity’*,* ‘Worry and guilt about work and milk’*, and *‘Sometimes it was less challenging’.* Themes presented rich, detailed accounts of both contextual and structural challenges faced by breastfeeding employees from this large Australian metropolitan hospital.

**Conclusion:**

Despite state and local health department policy that stipulates breastfeeding is to be ‘promoted, protected, and supported’, this study highlights the many barriers faced by hospital employees wanting to meet the health service’s own breastfeeding recommendations. These findings raise issues of gender equity in the health workplace and highlight the need for decent working conditions that actively support breastfeeding continuation after return to work in hospital environments. More broadly, findings point to the responsibility of hospitals to model best practice in supporting breastfeeding employees, aligning with public health recommendations and the rights of employees to combine paid work and breastfeeding.

## Background

The health and economic benefits of achieving the World Health Organization’s (WHO) breastfeeding recommendations are well understood [1]. However, around the world, structural and institutional barriers can hinder a mother’s ability to meet the WHO breastfeeding recommendations [2, 3]. The International Labour Organisation (ILO), which Australia is a signatory to, recommends that member countries adopt Conventions No.183 and No.191, ensuring access to maternity leave and the right to return to the same or equivalent work while breastfeeding [4–6]. ILO recommendations include a minimum of 14 weeks of paid maternity leave and designated breastfeeding/lactation breaks during working hours (4). However, internationally Return to Work Breastfeeding (RTWBF) practices vary significantly, influenced by national policies, cultural and economic conditions, as well as workplace environments [2, 3, 7]. Robust evidence shows that when workplaces implement protective conditions such as accessible space to pump, dedicated time, and policies that foster a culture of support, it enables RTWBF employees to achieve longer breastfeeding durations [2, 3, 7]. Yet despite the ILO recommendations, and the global evidence, gender inequalities persist, which adversely affects mothers’ ability to successfully RTWBF [2, 3].

Over the past four decades, female workforce participation in Australia has increased steadily from 40% in 1978 to 61.3% by 2019 [8]. This growth has been underpinned by the female-dominated healthcare and social assistance sectors, with most Australian women working through their childbearing years, albeit at reduced hours and pay [8–10]. Governmental support is available for Australian parents in permanent employment, in the form of 24 weeks paid parental leave (PPL), which has been increasing incrementally; by 2026 will increase to 26 weeks [4]. In addition, federal legislation protects the rights of Australian women, who RTWBF, however, marginalisation of breastfeeding employees remains commonly recounted [11–13]. Research by Xiang et al. (2016) found that returning to paid work before an infant reaches six months significantly reduces the likelihood of achieving the recommended six months of exclusive breastfeeding. Unfortunately, Australian mothers who return to work within 3 months of birth have a low probability of any breastfeeding by six months [10]. Furthermore, resuming paid full-time work within 10 months of birth negatively impacts “any breastfeeding” practices [10].

These findings are consistent with national breastfeeding data in Australia, which show substantial declines in breastfeeding over time with very few achieving the recommended breastfeeding duration and exclusivity time frames. Netting et al. (2022) reported in their national survey that 93% of Australian women commenced breastfeeding, but only 22% were still exclusively breastfeeding at 5–6 months [14]. Previously, the only National Infant Feeding Survey in Australia reported similar findings of 96% initiation rates of breastfeeding and 15% exclusive breastfeeding at 5 months [15]. Using both data sets, we can reveal that only 7–10% of Australian women in these reports reached the 2-year recommended milestone [15, 16].

In Australia, workplaces have the opportunity to bring their organisation up to the standard set by the Australian Breastfeeding Association (ABA) with Breastfeeding Friendly Workplace (BFW) accreditation [17]. While many working sectors have achieved BFW accreditation, including the New South Wales (NSW) State-based Ministry of Health head office (the Australian State where this research was conducted), uptake is low across hospital workplaces nationally [17]. Of approximately 697 Australian public and 657 private hospitals, less than 1% have reportedly achieved BFW accreditation [17, 18]. According to the ABA, only two NSW Local Health District has achieved BFW accreditation [17].

The experiences of Australian breastfeeding mothers who RTWBF have been examined across multiple workplace sectors, including female-dominated areas of teaching and nursing [7, 11], but there is a lack of contemporary evidence on Australian hospital employees’ experiences. The most recent Australian research, which focused specifically on hospital employees’ RTWBF experiences, was more than a decade ago [19]. This study aimed to explore the lived experiences of employees returning to work, from maternity leave, and maintaining breastfeeding, at one large metropolitan hospital in Sydney, NSW, Australia, which was not accredited as a BFW.

## Methods

### Study design

Interpretive Phenomenological Analysis (IPA) informed the methodology for this qualitative interview design. Creswell & Poth (2016) assert that a deeper understanding of human experience is available to the researcher through the IPA process by gathering a “contextual account” of what happened, and a “structural account” of how the experience happened. By adopting an IPA methodology, the focus was on the collection of data from participants’ lived experiences who RTWBF at this hospital [20, 21]. The analysis of the interview transcript included searching for themes across the data while also reporting on both structural and contextual accounts of the phenomena under investigation [21]. In this case, the seven-step IPA approach was used to gain insight into how employees perceived and managed the intersection of professional responsibilities and breastfeeding within the context of a hospital environment, reporting on structural enablers and barriers [22]. Purposive and snowball sampling were used to recruit employees who RTWBF at this hospital facility in NSW, Australia.

### Study site

This research was conducted in the Australian State of NSW, which operates more than 220 public hospitals and approximately 210 private hospitals [23]. At the time of data collection, the study site was the fifth-largest tertiary hospital in Australia, providing specialist health care, education, research, and health professional training [24]. The hospital had a 975-bed capacity, with approximately 5,000 permanent employees, a predominantly female workforce [25]. The maternity section of this hospital was a tertiary referral centre for high-risk infants and pregnancies and was not accredited with the WHO and UNICEF’s Baby Friendly Hospital Initiative (BFHI). The BFHI is a global program, available since 1991, which aims to ensure maternity facilities promote, protect, and support breastfeeding [26].

For the purposes of this study, breastfeeding was defined in accordance with WHO guidance to include any feeding of breastmilk, whether directly from the breast or expressed and provided by bottle, cup, or spoon [27].

### Ethics statement and consent to participate

Ethics approval was obtained from the facility-based human ethics committee (2021/ETH11153). Potential participants were provided with information about the study along with Human Ethics and Consent to Participate declarations. There was an opportunity provided to participants to ask questions before consenting to participate. Potential participants were informed that there would be no impact on their employment status or work relationships resulting from their participation and that they could withdraw at any time without reason or penalty. All participants were offered the option to have follow-up Employee Assistance Counselling (EAC) or ABA support if required. No changes were deemed appropriate after piloting the interview prompts and questions.

### Inclusion criteria

Employees were invited to participate in this study from all hospital departments: medical, nursing, midwifery, allied health, domestic, engineering, and clerical staff. Participants met the inclusion criteria if they self-identified as breastfeeding employees, were 18 years or older, and had RTWBF at the study site over the past four years. Participants had to be willing to participate in an in-depth one-on-one interview and able to freely provide consent. Employees who RTWBF at another hospital were excluded from this research. Staff who were unable to read or converse in fluent English or make informed consent were also unable to participate.

### Recruitment and data collection

A flyer advertising the study was posted across a range of ethics-approved areas. A QR code on the flyer took participants to the Participant Information Sheet (PIS) and landing page to leave their best contact details. Snowball sampling was also used to enhance the reach of the call for participants.

Data collection occurred from March to May 2021. As this was during Covid-19 pandemic conditions, all interviews were conducted using the online Zoom platform. Interviews were audio-recorded following confirmation of informed consent. The first author conducted 11 interviews, and a further two were conducted by the third author as participants were known to the first author. In-depth interviews commenced with the collection of demographic information prior to questions focused on the experiences of breastfeeding employees. The in-depth exploration of the participant experience was informed by the IPA approach and commenced with open-ended questions to allow the participant to control how much or how little of their story they would like to disclose. Active listening was used to ensure important comments were expanded upon. Interviews were scheduled for one hour and proceeded at the participant’s pace. Gentle prompting was used at times to enable the participant to expand the narration, with key prompts such as “*tell me about your experiences of RTWBF at this hospital”* and *“please expand on how this experience made you feel”.* Interview guides and key prompts were pilot tested by the research team before us. No remunerations were offered to participants, and interviews were conducted during working hours. Participants were offered the option to review their interview transcripts up to one week after the interview. None elected to undertake transcript review. For confidentiality purposes, all participants were allocated pseudonyms. (See Table 1).

**Table 1:** Demographic details of participants

Pseudonym	Age	Work area	Currently breastfeeding Y/*N*	Currently expressing at work Y/*N*	Employment	Shift work Y/*N*
Karen	30	NM	Y	Y	Full-time	N
Anna	36	NM	N	N	Full-time	N
Louise	40	AH	N	N	Part-time	N
Frances	37	HA	N	N	Part-time	N
Rosemary	32	AH	Y	Y	Part-time	N
Sheila	38	MP	Y	Y	Full-time	Y
Arpita	29	NM	N	N	Part-time	Y
Helen	32	MP	N	N	Full-time	Y
Evelynn	34	MP	N	N	Full-time	Y
Julia	33	NM	N	N	Part-time	Y
Terumi	42	NM	N	N	Part-time	N
Allison	35	NM	N	N	Part-time	Y
Maeve	33	MP	N	N	Part-time	Y

Legend: NM = Nursing & Midwifery; AH = Allied Health; MP = Medical Practitioner; HA = Health Administrator

### Data analysis

The first author analysed the data using the seven-step IPA analysis process, described by Smith & Osborn (2008). The in-depth analysis followed the recommended steps of 1- close reading and re-reading of each transcript; 2- initial noting of descriptive, linguistic, and conceptual comments; 3- development of emergent themes from within each transcript; 4- exploration of connections across emergent themes; 5- identification of shared patterns across cases while preserving idiographic details 6- identifying patterns across cases, and finally, 7- crafting the narrative to document the findings [28]. NVivo software was utilised to code the data into categories, subcategories, and then into themes and sub-themes. Codes and themes were developed iteratively, grounded in the participants’ narratives. The analysis took into consideration participants’ social positioning as self-identified female hospital employees navigating RTWBF, acknowledging the organisational and cultural contexts shaping their experiences. This was done by reading the transcripts several times and paying attention to how language showed gender expectations, professional hierarchies, and institutional power. This IPA process enabled analysis of each individual’s narration of their lived experience and how they made sense of their personal and social worlds [29].

### Reflexivity and rigour

The research team engaged in reflexive discussions to consider how their own positionality, including gender identification, disciplinary background, and professional experience, might influence questioning and reporting of participants’ accounts. Field notes were kept by the interviewers throughout the interview data collection and analysis stages of the project and discussed amongst the research team to avoid potential bias and ensure participants’ experiences were prioritised. The insider/outsider positioning of the researchers in this study was recognised as both an asset and a challenge [30]. As the team shared professional language, cultural knowledge, and organisational context with participants, which helped when building rapport during interviews, but this shared familiarity also introduced the risk of assumptions and preconceptions, particularly around workplace dynamics and breastfeeding support. Rather than removing researcher influence, the process of reflexivity involved an ongoing, critical examination of the researcher’s position. Additionally, when interpreting themes related to the labour of caregiving roles, the team discussed whether interpretations were grounded in participants’ narratives or shaped by societal assumptions about women in healthcare.

## Results

**Fig. 1:**
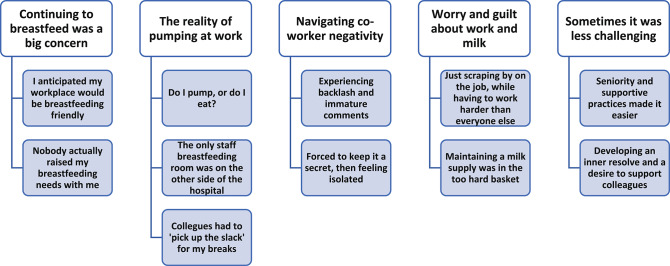
Themes and sub-themes

In total, 38 employees responded to the flyer advertisement. After filtering out employees who were non-contactable, unavailable for an interview, or fell outside the study criteria, 13 employees were left. Rich data was collected from all participants, with recurring themes becoming apparent after the first couple of interviews, and no new data arising by the thirteenth interview. A broad sample of employee roles was represented, including nursing/midwifery (NM) (*n* = 6); medical (MP) (*n* = 4); allied health (AH) (*n* = 2); and health administration (HA) (*n* = 1). Participants were aged between 29 and 42 years, and at the time of the interview, worked at the study site. Participants worked either full-time (*n* = 5) or on reduced or part-time hours (*n* = 8). There was a similar spread of shift-working employees (*n* = 7) and non-shift working participants (*n* = 6). Analysis of interview data revealed rich, detailed experiences. Common threads and patterns in the data were developed into five themes. Themes reflected the lived experience of employees, comprising: *‘Continuing to breastfeed was a big concern’*; *‘The reality of pumping at work’; ‘Navigating co-worker negativity’; ‘Worry and guilt about work and milk’*; and ‘*Sometimes it was less challenging’*. Each theme had related subthemes. (See Fig. 1).

### Continuing to breastfeed was a big concern

All participants reported an aspiration to continue breastfeeding upon returning to work but described feeling apprehension. There were concerns raised by most participants regarding the invisibility of their breastfeeding intentions and needs, with no accounts of RTWBF discussions with managers before maternity leave.

As Louise, an Allied Health participant, said, *“So*,* wanting to continue breastfeeding was a big concern for me going back to work”*, highlighting worry about meeting personal breastfeeding goals and the organisation’s willingness to accommodate her RTWBF needs.

### I anticipated my workplace would be breastfeeding-friendly

Participants expected their workplace, as a health facility with a comprehensive breastfeeding policy directive, would be breastfeeding-friendly. Helen, a doctor, said she expected support from her department to maintain her milk supply, but sadly found negligible facilities, processes, and provisions from the beginning of her RTWBF journey.

*I just hoped that I could keep going* [breastfeeding] *with quite a supportive department*,* and I thought like it would be taken into the planning of hospitals more about where staff are going to be able to do things like pump* (Helen, MP).

One nurse, Julia, was asked by her manager to cut her maternity leave short to help with poor staffing during the COVID-19 pandemic. She was assured that her RTWBF needs would be accommodated, but unfortunately, they were ignored. *I came back to work early*,* and*,* I guess*,* COVID times*,* I could see the strain and stress on my colleagues*,* but when I realised that there was just little to no support around this* [expressing at work] *at all*,* I was upset and really regretted not having a longer maternity leave* (Julia, NM).

Julia deeply regretted her decision to return early, as it contributed to her premature cessation of breastfeeding. Her experience underscores the emotional toll of unmet organisational promises and the serious implications of inadequate RTWBF support.

### Nobody actually raised my breastfeeding needs with me

Poor communication regarding RTWBF needs was frequently recounted, compounding a non-breastfeeding-friendly environment. Louise, an allied health professional, shared *“… no one raised breastfeeding with me*” (Louise AH), and Frances, an Administrator, said, *“At no stage did they ask me if I was breastfeeding”* (Frances, HA).

Managers were frequently seen to be either unaware of workplace entitlements or to display complete disinterest in accommodating RTWBF needs.

*I definitely would’ve wanted one of my managers to say*,* ‘When you come back and if you are expressing*,* you will be able to have this amount of time to do that. We will have a room for you*,* or we’ll direct you where to go* (Terumi, NM).

Most participants said that at the time they RTWBF, they were unaware of workplace entitlements, so were left to manage their own expressing breaks and provision.

### The reality of pumping at work

The real-life experience for many RTWBF employees was a day-to-day striving to find a safe place and time or an entitled break to pump. An allied health participant said it was stressful because she had to proactively arrange her own provisions. *“How do I manage this?’ I was trying to*,* I guess*,* manage my pumping at work and not get stressed”* (Louise, AH).

One doctor said, *“It was up to me to manage*” (Maeve MP). She went on to say self-managing, expressing time at work meant there were many days she had to forgo pumping altogether. Other participants said they relied on the goodwill of colleagues to cover for pump breaks, but for most, multitasking on meal breaks was the only option.

### Do i pump, or do i eat?

A nurse explained, her manager gave her limited choice when trying to maintain her supply.

*My manager at the time said*,* ‘You can pump or eat – your choice’* (Terumi, NM).

Arpita, another nurse, described the complexities of trying to juggle eating her meal and expressing milk for her baby meant she never had time to do either adequately. For some doctors, sporadic pumping between surgical cases and going without lunch was the only way to maintain a milk supply at work.

### The only staff breastfeeding room was on the other side of the hospital

Although one staff lactation room existed at this facility, participants reported the single-occupancy room was hard to find and a long distance from most areas.

*The problem I had was that the only breastfeeding room was on the other side of the hospital and quite difficult to find and get there. So*,* having my patient load on top of trying to eat lunch or morning tea and pumping at the same time*,* I really struggled* (Karen, NM).

An allied health participant said, “…*it was an expedition to get there* [breastfeeding room]” (Rosemary AH). Another nurse explained that when she eventually found the single-occupancy room, it was already being used. She was forced to return to her ward area and asked her manager to use ward space to pump breastmilk. Her manager disregarded her needs and denied her time or space.

Struggling to find adequate and convenient space to pump at work, for some, meant using patient areas and risking disapproval from managers. As Allison, a nurse, explained, “*I was using just a couple of rooms on the ward*,* which I don’t know that the main manager was happy about*,* but I just snuck in and used it anyway.”*

These accounts illustrate not only the lack of infrastructure but also the pressure to conceal breastfeeding-related needs to avoid conflict or reprimand, further contributing to a culture of silence around workplace lactation support.

### Colleagues had to ‘pick up the slack’ for my breaks

Self-managing expressing at work meant for some relying on co-workers to cover them with breaks, but this was not regularly achievable as certain co-workers reportedly refused to support RTWBF entitled breaks. *“But then I think your colleagues have to pick up the slack*,* really. Some don’t like that. You still need someone to look out for your patients. Otherwise*,* how can you go?”* (Anna, NM).

Another nurse explained she was reluctant to ask certain co-workers to cover for her, fearing negative repercussions. Anxious about maintaining her milk supply, she said she was forced to relieve the pressure in her breasts by hand expressing during a five-minute bathroom break and discarding her milk in the toilet. Another nurse discussed that without any breaks, some are forced to leave the working area, without cover for patients, due to breasts that were full, uncomfortable, and/or leaking.

### Navigating co-worker negativity

NSW Health employees are expected to provide a supportive environment for RTWBF staff and inpatients. Unfortunately, there were frequent accounts of negativity and likely discrimination from co-workers. As Arpita a nurse, shared, *“…some just made me feel really uncomfortable to the point when on shift with them*,* I would actually not even express.”* This experience reflects the pressure many participants felt to hide their breastfeeding needs in the face of unsupportive attitudes.

### Experiencing backlash and immature comments

Poor conduct from co-workers was described by Terumi a nurse as “*backlash and immature comments about breastfeeding”* (Terumi, NM). Subtle and overt discrimination forced many participants to push through without an adequate expressing break. Others kept their breastfeeding and expressing status a secret.

One nurse explained her co-worker’s negative behaviour culminated in the deliberate discarding of her expressed milk from the staff room refrigerator. The mistreatment of RTWBF employees was described by another participant as colleagues who “*exuded negativity towards my breastfeeding”* (Frances, HA). Whilst some participants described *“dreading”* coming to work, because certain co-workers disregarded RTWBF needs and displayed disapproval when requesting entitled breaks.

### Forced to keep it a secret, then feeling isolated

Negative and inaccurate opinions from co-workers regarding the duration of breastfeeding forced some to make the difficult decision to keep their breastfeeding status a secret. However, this often left them feeling isolated at work. Frances, an administrator, disclosed the following feelings of isolation, describing it as a lonely time.

*“When you are locked away in your office and you’re not socialising with your co-workers*,* because you are using your lunch break to do something that you really ought to be paid to do* [express], *it’s a lonely time”* (Frances, HA). This sense of isolation brings attention to the broader emotional toll of working in an environment where breastfeeding needs are marginalised rather than integrated into everyday workplace practices.

### Worry and guilt about work and milk

There were frequent accounts of RTWBF participants struggling to balance work demands and pumping. Helen, a doctor, explained daily challenges and feelings of worry and guilt, trying to regain work confidence in an unsupportive breastfeeding environment while providing milk for her baby. Another participant, a nurse, said the daily worry about finding time to pump provoked conflicting emotions for her. She questioned whether it was too hard to continue.

*So*,* yeah*,* you’re just mixed up because you’re like*,* ‘Do I bother* [expressing]?*’ but I felt guilty then about my girl*,* but the stress of it all and the worry of if I am going to be ok to do it* [pump] *today* (Allison, NM).

This poignant statement captures the emotional burden many participants faced—torn between their commitment to their infant’s needs while navigating the practical challenges of an unsupportive work environment.

#### Just scraping by on the job while having to work harder than anyone else

Participants described the struggle associated with balancing two important priorities: milk production and work demands. Some described feeling inadequate at both and having to work harder than anyone else to keep up. Louise, an allied health professional, described it as: *“Just scraping by on the job*,* I feel dumb with all the bright sparks running alongside me with time to keep up”* (Louise, AH).

In an environment without support for their breastfeeding needs, participants commonly said they had double the workload to keep up. Frances, a health administrator, described how she felt she had to reestablish herself and work harder than anyone else to prove herself capable after maternity leave. Evelynn, a doctor, said: *“I would’ve had to cancel clinics*,* made waiting lists even longer if I didn’t keep up. I don’t think they could ever say I didn’t work hard enough”* (Evelynn, MP).

Putting in more working hours to compensate for expressing breaks was a frequent strategy. An allied health participant explained she had to work after hours to keep up and had no alternative but to fake the figures. She disclosed needing to lie to colleagues and managers about the time she spent with patients to cover up the time she spent pumping milk for her baby.

#### Maintaining a milk supply was in the too-hard basket

Regular expressing at work was reportedly unachievable for most, which ultimately resulted in unintentional breastfeeding cessation. Without workplace support or knowledge of workplace entitlement, Anna, a nurse, said she was forced to prematurely stop breastfeeding. *“I had to put it* [breastfeeding] *in the too-hard basket”* (Anna, NM).

Early, unintentional loss of breastfeeding left Anna feeling deeply unhappy.

*I felt quite defeated and guilty that I’d worked so hard to maintain this breastfeeding relationship that we struggled to begin with and worked really hard to establish*,* and then coming back* [to work] *it just all fell to pieces*,* a lot sooner than I was planning it to* (Anna, NM).

One nurse said the lack of expressing rooms or time to express at work affected her supply, but experiencing negativity from co-workers was the ultimate straw that forced her to cease breastfeeding. Without a network of support, marginalisation of RTWBF employees was commonly reported. As Helen, a doctor, said, *“There wasn’t much of a support network. It was too difficult to keep going because everybody before had found it difficult to maintain. So*,* it just ended quite quickly for everybody*,* and nobody really spoke about it after that”* (Helen, MP).

Helens’ account illuminates the pervasive sense of isolation and silence surrounding breastfeeding challenges in the workplace. This lack of a support network not only hinders individual breastfeeding continuation but also perpetuates a culture where these issues remain unaddressed.

### Sometimes it was less challenging

Two participants described fewer challenges. These employees were less reliant on managers or were in a clinical/management role themselves. In addition to seniority, enablers included supportive colleagues or developing an inner resolve and self-confidence.

#### Seniority and supportive practices made it easier

Conscious of their place professionally, two participants, who held senior jobs, discussed how their position facilitated expressing at work. Shiela, a senior doctor, said:

*I was lucky that I could have registrars go and see the patients for me in the main clinics. My private clinic was a bit harder*,* but again*,* it was probably easier to allocate the time to express in between patients* (Sheila, MP).

Supportive practices offered a snapshot into the differences that an enabling workplace breastfeeding culture makes. Without this support, Sheila said it would not have been that easy for her to continue.

*My boss was incredibly supportive*,* as well as everyone in the office. So*,* it was easy. Everyone just laughed at me when I was expressing at my desk. But yeah*,* I don’t think if it hadn’t been such a supportive environment*,* it would’ve been as easy* (Sheila, MP).

Regular working hours and a supportive manager enabled Rosemary, a senior allied health participant, to schedule her workdays to self-regulate her meal break times. Having her own private office space enabled privacy to express; however, she often multitasked, combining office work, pumping, and eating.

#### Developing an inner resolve and a desire to support colleagues

Some participants described developing an inner resolve and self-confidence despite the many workplace barriers. *“I just felt like I’m not going to give that up regardless of who accepts it or not. For me*,* it’s like my duty for my kids”* (Terumi, NM).

Understanding the barriers and hardships faced, many participants expressed a strong desire to help and support other RTWBF colleagues. As Allison, a nurse, and Maeve, a doctor, explained, they shared their knowledge and experiences with other breastfeeding colleagues in the hope of making it easier for them. A senior doctor, Shiela, talked about how important it was to set an example for junior staff by advocating for breastfeeding peers. While Francis, a health administrator, said she made a point of discussing workplace entitlement with her co-workers who were going on maternity leave. She explained that a conversation regarding RTWBF options should be part of a resuming maternity leave package.

## Discussion

To our knowledge, this is the first Australian study, since 2011, to specifically examine hospital employees’ experiences of returning to work while breastfeeding. Thirteen hospital employees contributed experiential data for this qualitative research, describing a predominantly unsupportive workplace culture with negligible facility processes and provisions for RTWBF hospital employees. These findings highlight a gap between the health department’s breastfeeding policy and actual hospital practices [31]. Although this hospital facility has not achieved the Baby Friendly Hospital Initiative (BFHI) accreditation, Ministry of Health policy directives stipulate that this, and all hospital environments, should ‘promote, protect and support’ breastfeeding and operate as breastfeeding-friendly workplaces [26, 31]. Additional facility guidelines further reinforce that staff who are expressing breastmilk or breastfeeding should have breaks and an accessible space as part of their workplace rights [24].

This study revealed a lack of awareness among employees interviewed, their co-workers, and managers about breastfeeding rights, resulting in unsupportive practices. The absence of accessible, appropriate spaces to breastfeed or express milk presented an unnecessary barrier and hardship. Additionally, findings point to gender inequalities and discrimination, where these RTWBF hospital employees were marginalised by managers, coworkers, and the organisation.

### Managers and employees lacked awareness of breastfeeding rights

Many participants from this Australian study reported being unaware of their workplace entitlements or rights. This caused considerable stress and hardship, rendering them unable to advocate for themselves. Although both state and local policies explicitly outline the rights of RTWBF [24, 31], participants described encountering resistance from managers and colleagues who appeared to be unaware that breastfeeding or expressing breastmilk at work is a protected entitlement. Restricting these rights may constitute discrimination or be deemed unlawful [12, 13, 32].

This general lack of awareness of breastfeeding workplace rights aligns with a systematic review by Dinour and Szaro (2017), highlighting the importance of workplace policy and structures that uphold breastfeeding entitlement. Similarly, a recent large Australian workplace survey found a key predictor in maintaining milk supply after RTWBF was when employees had the confidence to stand up for their rights and had a suitable place to express milk or breastfeed. Essentially, awareness of entitlements and access to space increases RTWBF employee confidence, and the odds of meeting breastfeeding goals [11].

Addressing the needs of RTWBF employees in a 24-hour healthcare environment can undoubtedly be complex for health managers. Nevertheless, strong evidence highlights that both facility and managerial-level support for breastfeeding employees are critical [33, 34]. Froh and Spatz (2016) showed that even when American workplaces have lactation programmes, RTWBF needs can be overlooked by managers and co-workers. However, when managerial-level support is combined with formal facility RTWBF policy and provisions, breastfeeding outcomes are improved [34, 35]. Managerial support and knowledge of RTWBF entitlement increase job satisfaction as well as the likelihood of achieving breastfeeding goals [34]. By communicating support and accommodating RTWBF employees’ needs, managers not only strengthen the facility’s breastfeeding culture but model best practice, setting a positive example for other staff [33, 34, 36, 37].

### Hospitals need accessible, appropriate space for breastfeeding employees

Our study revealed a critical shortage of accessible, dedicated spaces for expressing breastmilk, sending a message of organisational indifference toward breastfeeding employees. This finding reflects both Australian and international research, which consistently shows that accessible, suitable breastfeeding or expressing space at work is a key predictor of breastfeeding continuation and, importantly, a visible marker of whether a workplace is supportive or not [2, 7, 35]. For hospital employees, the challenges can be amplified with shift work, unpredictable schedules, and workloads [35, 38]. Henry-Moss et al. (2019) in the US made recommendations to have expressing spaces within a 5–6-minute walk to accommodate working day realities for hospital staff.

The challenges reported by women in this qualitative study mirror international evidence, where RTWBF hospital employees similarly faced RTWBF challenges of inadequate facilities [37, 39–44]. Given that the WHO’s *Gender Equity in the Health Workforce Analysis* (2019) shows women make up 70% of the global health workforce [45], failing to accommodate breastfeeding employees could risk health workforce wellbeing, recruitment, and retention [34]. In addition, this lack of workplace support could undermine workforce capacity and compromise economic productivity [34, 46]. Australian researchers Smith et al. (2023) developed the ‘*Mothers Milk Tool’* to bring attention to the economic value of breastmilk and breastfeeding. The tool highlights the substantial quantities of human milk produced by mothers, and the negative economic consequences when mothers’ breastmilk and breastfeeding needs are dismissed [47]. Breastfeeding boosts economic productivity by supporting maternal-child health and reducing future health care and workforce costs; therefore, investing in workplace support and policies for RTWBF employees is a smart economic strategy [46–48].

### Gender inequality in hospital workplaces marginalises employees

Despite being a predominantly female workforce, the findings suggest that this hospital operates within entrenched patriarchal norms [49, 50]. This was evident in how RTWBF needs were largely ignored or dismissed, possibly seen as a “private” or “personal” matter rather than a workplace responsibility [2]. Such attitudes reinforce the gendered divisions and marginalise hospital employees’ caregiving responsibilities [2, 7, 51–53], directly contradicting NSW Health workforce and patient care gender equity goals [51, 54].

These patterns mirror broader gender inequalities observed across other female-dominated industries in Australia, despite legal protections prohibiting discrimination based on breastfeeding or expressing breastmilk [11–13, 55]. Australian researchers Ayton et al. (2025) discussed how workplace planning and policy often neglect the intersection of breastfeeding, paid employment, and family life, thereby reinforcing gender inequality and placing the responsibility for managing RTWBF largely on mothers themselves. American research draws attention to the contradiction inherent in hospital workplaces built on health and caring that fail to recognise or provide support for their own employees [41]. For meaningful progress, policy-makers and health systems must acknowledge that the healthcare workforce is predominantly female, routinely subject to gender-based caregiving responsibilities, and therefore entitled to gender specific rights [41, 53].

### Protecting the breastfeeding rights of hospital employees matters

This qualitative research highlights the in-depth experiences of a number of RTWBF participants of this hospital, whose rights were largely disregarded or de-prioritised by the facility and coworkers. Zhuang et al. (2018) indicated, breastfeeding employees can often be incorrectly labelled as unprofessional or selfish by co-workers or managers, for taking time away from the workplace to express milk or even for wanting to continue to breastfeed. However, when early, unintentional breastfeeding cessation is forced upon mothers, it can have devastating consequences on mothers’ emotional and psychological well-being [56]. Feelings of prolonged grief and failure have been described in Australian literature as a *“deep and penetrating sense of guilt and shame”*, with the unintentional loss of breastfeeding [56]. Villar-Comte and colleagues (2022) recommend that promoting, protecting, and supporting breastfeeding should be seen through a social justice and health equity lens; therefore, impeding or not recognising the right to breastfeed should be viewed as a human rights violation [57].

Being prevented from achieving the health services’ own breastfeeding goals could be particularly problematic for the hospital workforce. Dixit et al. (2015) recounted the feelings of paediatric trainees, who, acutely aware of the impact of not meeting breastfeeding goals, disclosed their RTWBF experience to be “*frustrating*,* devastating*,* and depressing*” as a result of a disabling workplace. Similarly, Turkish and American findings demonstrate that hospital employees experienced feelings of stress, guilt, and even poor bonding with their children as a result of forced, unintentional breastfeeding cessation due to workplace neglect [58, 59]. Compounding the issues reported, health professionals’ personal breastfeeding experiences may directly shape their level of breastfeeding advocacy and support they provide to breastfeeding hospital inpatients.

### Personal experiences can influence health employees’ level of breastfeeding advocacy

Evidence shows that health professionals’ own breastfeeding experiences can affect how they interact with breastfeeding mothers in hospital. For example, a recent Irish study demonstrated that health employees’ personal experiences of breastfeeding establish a “personal breastfeeding philosophy” which influenced their knowledge and confidence when interacting with breastfeeding mothers [60]. Similarly, American researchers Sattari and colleagues (2013) found that one of the most effective interventions to increase breastfeeding initiation and duration for mothers is support and accurate advice from their physicians. Yet the literature highlights that the strongest predictor of physicians’ level of breastfeeding advocacy is their own personal experiences, or their partners’ breastfeeding experiences [61]. Further supporting this connection, Dixit and colleagues (2015) found that a disabling workplace for RTWBF paediatric interns impacted how these doctors engaged with and supported breastfeeding patients. Notably, 92% of participating paediatric interns disclosed negative experiences with their own RTWBF experiences [62].

As previously mentioned, this study site was not BFHI-accredited. Across Australia, only 23% of facilities have achieved accreditation, despite strong evidence indicating BFHI accreditation positively influences breastfeeding rates at hospital discharge [63, 64]. An accredited BFHI facility also increases employees’ breastfeeding knowledge and confidence [63, 64], yet to our knowledge, there is a lack of evidence on employees’ RTWBF experiences in a BFHI-accredited hospital.

Findings from this Australian research add to the body of international knowledge and bring attention to an urgent need for hospital systems and governments to scale up protection, promotion, and support for RTWBF hospital employees. These results position RTWBF support for hospital employees, not merely as a workplace right, but as a strategic investment in workforce sustainability, gender equity, and public health.

### Strengths and limitations

To our knowledge, no other study has explored Australian hospital employees’ perspectives on this issue in more than 15 years. This makes the findings a unique and timely contribution to the field, which is a strength. The use of in-depth interviews enabled the collection of rich, detailed, and meaningful data. Strengths also include the inclusion of employees from multiple hospital professional groups.

This study has several limitations. First, while in-depth interviews provided rich insights, the absence of triangulation could reduce the rigour of the findings. Secondly, the reliance on self-reported accounts introduces the possibility of recall bias and socially desirable responses. Third, the study was conducted with a small sample of 13 hospital employees from a single site, which limits the transferability of findings to other contexts. Despite these limitations, the findings are similar to broader literature, which enhances their credibility and relevance.

## Conclusion

This study offers important insights into the experiences of Australian hospital employees who RTWBF. Despite national and international policy frameworks intended to protect the rights of breastfeeding employees, participants from this Australian hospital described a lack of structural support, managerial indifference, and co-worker discrimination. Barriers to equitable breaks were common, with a serious shortage of space to express breastmilk. The consequences of an unsupportive workplace breastfeeding culture impact breastfeeding duration and are associated with poorer health outcomes for infants, mothers, families, and society. An unsupportive breastfeeding culture has implications for staff well-being, productivity, and attrition. Importantly, advocacy for breastfeeding may be undermined by hospital employees’ own negative experiences during their RTWBF journey. These findings expose marginalisation of women’s caring responsibilities and gender inequities in the hospital workplace, highlighting a critical disconnect between hospital policy and actual practice. Findings align with the ongoing undermining of the gendered nature of breastfeeding, despite its recognised health, economic, and social value. Results point to a pressing need for this hospital and all hospital facilities to prioritise the protection of RTWBF employee rights, not only as a legal or ethical obligation but as a strategic imperative for a thriving, inclusive, and sustainable health workforce.

## Data Availability

The transcripts and generated analyses of the study are not publicly available due to confidentiality and anonymity agreements.
